# The Effects of a Peer-Counseling Program on Increase Rate and Continuity of Lactation in Tehran Nursing Mothers 

**Published:** 2019-12

**Authors:** Fatemeh Nayeri, Nahid Farrokhzad, Tahereh Esmaeilnia, Nikoo Niknafs, Hossein Dalili, Lida Atarod, Hasti Charousaei, Mamak Shariat

**Affiliations:** 1 Breastfeeding Research Center, Institute of Family Health, Tehran University of Medical Sciences, Tehran, Iran; 2 Maternal, Fetal and Neonatal Research Center, Institute of Family Health, Tehran University of Medical Sciences, Tehran, Iran; 3 Department of Pediatrics, Imam Khomeini Hospital Complex, Tehran University of Medical Sciences, Tehran, Iran

**Keywords:** Lactation, Exclusive Breastfeeding, Counseling

## Abstract

**Objective:** The practice of breastfeeding is considered a blessing since its effects on health are well recognized and applies to both mothers and infants. The objective of this study was to evaluate the effectiveness of peer support and training on breastfeeding initiation, duration and exclusivity.

**Materials and methods:** This community-based clinical trial, (IRCT No: 201504049568N12), was conducted during 2015 in the Municipality of Tehran 19 District. First, a total of 150 mothers with their infants from 4 to 20 months of age were asked to complete a questionnaire, which included the demographic characteristics, educational level, and the type of lactation, the initial age of infant for breastfeeding, and the duration of exclusive breastfeeding. Afterwards, 25 volunteer women were selected for lactation counseling. After 6 months, another sample of 116 nursing mothers in the region who had received peer counseling was selected and questioned through the previously mentioned questionnaire. Finally, the results, which were collected from the behavior of the target population before and after the intervention, were compared.

**Results:** The results of the present study indicated that the nursing mothers who received peer counseling proved to have longer durations of breastfeeding (P-value = 0.039), and higher frequency of first hour initiation of breastfeeding (P-value = 0.003) however, the volunteer counselors were mainly housewives who had lower levels of education (P-value = 0.009) and were younger (P-value = 0.009) than those of untrained control group.

**Conclusion:** The study demonstrated the significant effect of peer counseling on breastfeeding initiation and continuation. It is suggested that lactation training could be initiated during the prenatal period along with the conventional methods of training.

## Introduction

The practice of breastfeeding is regarded as a blessing since its effects on health are well recognized and applies to both mothers and infants (1). The healthcare personnel and the mass media have taken a host for significant measurements in Iran so far for raising awareness and empowering mothers. Promoting of breastfeeding for instance has been one of the priorities of the Ministry of Health and Medical Education in Iran over the last several years. Thus, the healthcare personnel including the General physicians and Midwifes, Gynecologists, Pediatricians and Neonatologists are required to attend and complete the ministry's breastfeeding workshops. Moreover, the NGO of IRI Breastfeeding Promotion Society has pursued extended educational and research activities. The media in general and the radio and television in particular have implemented policies towards publicizing and promoting the practice of breastfeeding in the society. Hospitals are also obligated to hold classes and workshops about pregnancy and preterm duration that present various and fruitful information tips and techniques, benefits and complications on breastfeeding (2). As the result of mentioned approaches above, there has been a growing trend towards breastfeeding practice throughout the country; however, this trend has fluctuated over the past 10 years in some urban and rural areas in different provinces. That could be caused by variety of factures, for instance the trainees might find the breastfeeding education course ineffective since during pregnancy the issues of lactation and breastfeeding are not tangible due to their lack of actual lactation experience; as a result, many potential questions have yet to occur for them. In addition, after delivery or during recovery, general conditions do not allow new mothers to concentrate sufficiently on education. Hence, the training provided in the Postpartum Unit by midwives is not effective or fruitful. However, after discharging from the hospital and initiation of lactation, and even in the following months, mothers have many needs and encounter a number of problems and questions when physicians, nurses and health care personnel are neither always nor easily available (3). According to the Iranian Ministry of Health and Medical Education, the percentage of children who are neither breastfed nor frequently breastfed is 83/93% (4), which is significant.

The results of the studies in other countries also support the fact that provision of breastfeeding education by trained mothers is more effective than the healthcare personnel (5). 

Another study in the United States in 2004 also reported the outstanding impact of peer- counseling on initiation and continuation of breastfeeding among nursing mothers (6). Peer-consolation not only improves and increases initiation and continuation of breastfeeding rates but also decreases diarrhea in newborns and increases the duration of amenorrhea in the mothers (7).

Nowadays the Municipality of Tehran, have made trustworthy and appropriate health resources and services for pregnant and breastfeeding mothers. Health Houses for instance, have been established in each and every neighborhood consisting of various departments and centers such as Mother and Child Centers. They are aimed for active involving community healthcare workers to provide health and social services. In the present study, we gathered a number of trained mothers (supervised by healthcare helpers and trained educators) to counsel and train other nursing mothers and offer them help and support in order to evaluate the effectiveness of peer-support and training on breastfeeding initiation, duration and exclusivity comparing to the conventional approaches according to the sociocultural conditions of Iranian society.

## Materials and methods

This study was conducted from January 2015 to January 2016 with the support of the health centers in 19 district of Tehran Municipality.

This community-based clinical trial was approved by Ethical Board committee of Tehran University of Medical Sciences with the number 93-04-105-27808-143712 and was registered at IRCT with the number 201504049568N12.

The participants included nursing mothers with infants up to 20 months of age who were living in 19 district of Tehran Municipality. 

Exclusion criteria included Mothers who were unable to breastfeed for any reason, mothers who moved out of 19 district of Tehran Municipality during the study, as well as mothers who didn’t wish to participate, prior to completion of data gathering were excluded from the study. A number of 133 nursing mothers before and 116 mothers after receiving the intervention were studied.

First, a total 25 volunteers of healthcare helpers at Mother and Child Centers in the Municipality of Tehran 19 District participated in a 2-day workshop course, which was held by experienced faculty members and lactation experts. In this workshop, with an easy to understand language, the importance of maternal breastfeeding and its improvement methods including the initiation of breastfeeding in the first hours, exclusive feeding, breastfeeding during infant and mother’s illnesses, and etc. was taught.

Additional training was also provided by paper and electronic media after ending of the course. Next, these trained healthcare helpers evaluated the breastfeeding initiation rates, and its continuation as well as the associated factors with early discontinuation of breastfeeding for the mothers who had infants from 4 to 20 months of age, and were living in 19 district of Tehran. The evaluation was carried out through a pre-designed questionnaire provided to the healthcare helpers. This group was considered as the control group or the pre-invention group. The trained healthcare helpers then formed educational committees of both peer and volunteer nursing mothers and trained them with the help and supervision of the mentioned faculty members and lactation experts. By the end of the one-month course, this new group of mothers gained the required skills, and were able to act as peer-counselors and mentors in the mentioned area. At each stage of training in this area, the group had the opportunity to be easily in contact with the faculty members and consultants of the Breastfeeding Research Center at specific times, in person or by telephone, and also could refer cases that required treatment or interventions. Finally, the group of trained volunteers began to collaborate with the study to educate and provide advice to nursing mothers in their own area, supervised by healthcare helpers and members of the research center.

Six months after receiving the intervention, by studying eligible mothers who were consulted during breastfeeding, the status of the research environment was again assessed and evaluated. This group received the intervention and was called the interventional group. 

The evaluation process before and after the intervention was carried out through a questionnaire. The questionnaire included variables such as maternal general and demographic information, midwifery records, records of involvement in neonatal care training, neonatal information, age of breastfeeding initiation, the lactation type (breastfeeding, formula, or mixed), duration of exclusive breastfeeding, and number of daily breastfeeding times (more or less than 8 times a day) (8).

The questionnaire was administrated to both groups and data was gathered and analyzed by SPSS version 18. The T-test for normal distributed and Mann Whitney for on-normal distribution were used for statistical data analysis of the study. A chi-square test was also used to determine whether proportions of variables differed from one another.

## Results

From 300 participants, 51 cases that moved out of 19 districts or didn’t wish to participate were excluded from the study. The exclusion criteria included 17 women before and 34 after carrying out the intervention. As a result, the number of participants in the pre-intervention group reduced to 133 and in the post-intervention group reduced to 116.

The educational level of the participants in the first and second group was one of the variables investigated in this study. As it is presented in table 1, participants of the pre-intervention group had higher levels of education (Table 1).

**Table 1:** Comparison of demographic information in both before and after intervention groups

**Scales**	**Before intervention group** **(n = 113)**	**After intervention group** **(n = 116)**	**P ** **value** +
Education	92(69%)	99(85%)	0.009*
Diploma and lower education [no. (%)]			
University education [no. (%)]	41(31%)	17(15%)	
Job			
Housewife [no. (%)]	106(80%)	107(93%)	0.002*
Employed [no. (%)]	27(20%)	9(7%)	
Mother's age when doing the study (mean ± SD)	31.15** ± **5.0	29.12 **±** 6.0	0.009**
Mother's age at marriage (mean ±SD)	21.08** ±** 3.8	20.05 **±** 3.8	0.009**
Delivery Type			
Normal vaginal delivery	53 (40%)	49 (42%)	0.140*
Cesarean section	80 (59%)	67 (57%)	
Complaints of postpartum depression	12 (9%)	13 (12%)	0.335*
Mother-infant separation	11 (8%)	6 (5%)	0.431*

+ Note. Statistical significant (p < 0.05),

* Independent T-test,

** Chi-square test

**Table 2:** Comparison of the frequency of first lactation and exclusive breastfeeding duration in both groups before and after intervention

**Scales**	**Before intervention group** **(n = 133)**	**After intervention group** **(n = 116)**	**P value** +
Breast feeding immediately after birth [No. (%)]	108(81%)	80(69%)	0.003*
Duration of exclusively breast feeding (mean ± SD)	9.41 ± 7.20	11.72 ± 80	0.039**

+ Note. Statistical significant (p < 0.05),

* chi-square test,

** independent T-test

Another determined variable was whether maternal employment was associated with the increase and continuity of lactation. As indicated Table 1, maternal employment had a negative effect on continuation of lactation in our study (Table 1).

Considering age as another variable in the study, it was observed that participants of the post-intervention group were both younger and had married earlier comparing to the pre-intervention group (Table 1).

After reviewing the first breastfeeding session in the pre and post intervention groups, a significant difference was observed. The mothers of the pre-intervention group who started lactation during the first hour after birth were more than the other group. According to Table 2, breastfeeding initiation rate was higher in the pre-intervention group comparing to the post-intervention group (81% vs. 69%, P-value = 0.03) (Table 2).

Analysis of duration of exclusive breastfeeding in the study revealed that its duration was considerably higher in the post-intervention group. In addition, 60% of mothers in the post-intervention group exclusively breastfed for six consecutive months after birth, while its number for the other group was 40% (P-value = 0.045) (Table 2).

Moreover, it was found that the number of mothers doing the breastfeeding practice more than 8 times a day (8), in the second group was more than the first group. However, its difference wasn’t significant (80% vs. 75%, P-value = 0.41).

No significant difference was observed between the prenatal and postnatal intervention groups in the type of delivery, complaints of postpartum depression and mother-infant separation (Table 1).

## Discussion

Since no prior research or study that shows the results of educational intervention of peer-counselors on lactation has been published in Iran, the results of the present study are of a great importance. What distinguishes the present study from other related studies is the fact that the present study is the combination of peer-counseling and community-oriented implementation.

The purpose of this study was to evaluate 1) duration and frequency of breastfeeding, 2) exclusive breastfeeding rate during the first six months after birth, and finally 3) the effectiveness of peer-counseling on promotion of breastfeeding initiation, duration and exclusivity, in our research environment.

The present study demonstrated that the rates of breastfeeding duration, frequency and exclusivity, in our research area, weren’t satisfactory before the implementation of intervention. However, these factors promoted after receiving the peer-consultation. The study also indicated that although the nursing mothers who were trained by the volunteer women were mostly housewives and also younger in age and were less educated than the untrained lactating mothers, after being educated, had longer breastfeeding duration, extended exclusive breastfeeding, and more frequent breastfeeding.

Based on the results of the present study together with the results of other studies in different parts of the world, it can be concluded that peer-counseling significantly affects breastfeeding initiation and continuation. In the studies by Chapman, a significant effect of peer-counseling on early lactation and continuation of exclusive breastfeeding was reported (5, 6). Findings of another study in Bangladesh indicated that the rate of breastfeeding in the first 5 months after birth was considerably higher in the intervention group who were consulted and trained by mothers of the same group (9). The results of it were in line with the reports of a study in southeastern America that showed an improvement in continuation of lactation of the groups who were trained by peer counselor women compared to the control groups, which was in agreement with our study (10). According to the results of the present study, despite young age and low levels of education, exclusive and continued breastfeeding will promote as long as mothers receive peer-counseling. 

In our study, nursing mothers who received counseling and advice from the volunteer women who had attended the breastfeeding courses, proved to have more frequent and longer breastfeeding duration and higher rates of exclusive breastfeeding as well, while they had lower levels of education comparing to the first group who received no training and peer-counseling. However, after receiving training they had longer lactation and exclusive breastfeeding plus more frequent breastfeeding. 

On the other hand, a study by Tarrant et al. in 2011 claimed that higher maternal education positively correlates with mother's breastfeeding at the time of discharge from the hospital and its continuation for the first six months after birth (11). The results are in line with another study by Oniwon, which indicated that higher maternal education is effective in increasing the rates of breastfeeding frequency and duration (12). 

The results of the present study reported that younger mothers proved to have extended breastfeeding duration, though some reports claimed that lactation increases with maternal age (13, 14). On the other hand, Lau et al. didn’t find any correlation between maternal age and breastfeeding duration (15).

***Limitation/recommendation:*** This study was carried out in one of the districts of Tehran, which was a limited scale; further studies can be conducted in each municipality district separately. Consequently, the volunteer women in each neighborhood after receiving the required training and understanding necessary solutions regarding their own neighborhood, can provide help as nursing mothers in the same area with extensive and long-term instructions. 

In addition, to make breastfeeding education more effective, as suggested it is better to start the educational interventions during pregnancy.

## Conclusion

The study demonstrated the significant effect of peer counseling on breastfeeding initiation and continuation. It is suggested that lactation training could be initiated during the prenatal period along with the conventional methods of training.
